# A novel two-step purification process for highly stable C-phycocyanin of analytical grade purity and its properties

**DOI:** 10.1007/s00253-025-13458-6

**Published:** 2025-03-24

**Authors:** Anna Antecka, Rafał Szeląg, Stanisław Ledakowicz

**Affiliations:** Department of Bioprocess Engineering, Faculty of Process and Environmental Engineering, Lodz University of Technology, Wolczanska 213, 93-005 Lodz, Poland

**Keywords:** Thermostable C-phycocyanin, pH and photostability, Two-step purification, Downstream processing, Analytical purity

## Abstract

**Abstract:**

Efficient and economic purification of phycobiliproteins can be achieved by a novel relatively simple two-step process involving foam fractionation and ion exchange chromatography. Foam fractionation, which has not previously been used to concentrate phycobiliproteins, is a low-cost and environmentally friendly method that provides a significant volume reduction prior to the chromatography step. Two C-phycocyanin fractions with purities of 4.66 and 4.25 with slightly different characteristics and an allophycocyanin fraction with a purity of 3.23 were obtained. Both C-phycocyanins contain α-subunits of 15.0 kDa and β-subunits of 16.4 kDa, whereas the molecular weight of allophycocyanin is 15.5 kDa. The resulting C-phycocyanin retains its properties at pH in the range of 3–10, whereas strong alkaline pH leads to its rapid degradation. The purified protein is completely resistant to temperature changes in the range of 4 to 50 °C and loses only about 13% of its initial concentration during a 5 h incubation at 60 °C. Interestingly, purified C-phycocyanin is relatively resistant to photochemical degradation, as the loss in concentration after 10 h exposure to light is only about 14%. The most suitable storage conditions are temperature of 4 °C and pH in the range 4–5. The final product with an analytical purity greater than 4 is suitable for use in food, biomedicine and as a therapeutic agent.

**Key points:**

• *Foam fractionation and ion chromatography for the purification of phycobiliproteins*.

• *C-phycocyanin stable over a wide temperature and pH range without a stabilizing agent*.

• *C-phycocyanin of analytical purity for food, medical and pharmaceutical applications*.

## Introduction

C-Phycocyanin (C-PC) is a pigment-protein complex found in cyanobacteria that biologically cooperates with chlorophyll in photosynthesis. Structurally, it belongs to the phycobiliproteins (PBPs), which are classified according to their colours as blue phycocyanin (C-PC), allophycocyanin (APC) and red phycoerythrin (PE) (Nascimento et al. 2020). C-PC is of great importance due to its various medical and pharmacological properties, e.g. anticancer, antioxidant and anti-inflammatory activities or potential use in the treatment of Alzheimer’s and Parkinson’s diseases (Piniella-Matamoros et al. 2021). They can also be used as natural colorants in food (Yu et al. 2024), cosmetics and pharmaceuticals (Eriksen 2008). However, due to the drawbacks of extraction and purification methods and the low stability of the compounds after the processes, the actual industrial application of C-PC is still limited (Pez Jaeschke et al. 2021). Furthermore, phycobiliproteins can command very high prices ranging from 3000 to 25,000 USD/kg depending on purity (Koller et al. 2014). Downstream processing (DSP), which accounts for up to 80% of the cost of bioproducts production, appears to be a key step worth exploring and optimising for a more sustainable production process. One very promising and under-researched DSP method is foam fractionation (FF), which belongs to the bubble separation technologies. Previous studies (Antecka et al. 2022, 2024) show that this method may be suitable for C-PC, as it is carried out under mild conditions and is suitable for dilute solutions. No less important, especially when assessing their use for industrial purposes, is the stability of the protein molecules, which is related to the appropriate control of process parameters, such as temperature, pH and ionic strength (Chaiklahan et al. 2011). Appropriate storage conditions for these fragile bioproducts, which guarantee the preservation of their valuable properties, are also key parameters to be considered. Most studies to date have focused on the use of additives to improve the stability of PBPs (Martelli et al. 2014). This seems to be the best and the simplest way, as it is easy to apply and does not require sophisticated or expensive equipment. However, additives with low toxicity should be used, as large amounts of additives may be required (Hsieh-Lo et al. 2019).

In general, C-PC in solution is a complex mixture of monomers, trimers, hexamers and other oligomers and its degradation depends on the aggregation state, which is influenced by parameters such as light, temperature, pH or protein concentration (Jespersen et al. 2005). Of the process parameters, the pH and temperature are the main factors controlling the aggregation and dissociation of C-PC and therefore play an important role in its stability (Chaiklahan et al. 2012). At pH 5, C-PC occurs predominantly in the hexameric form, and this form is thought to provide protection against denaturation. At pH above 7, it occurs mainly as a monomer or trimer, resulting in lower thermostability. It follows that an increase in pH above pI increases the solubility of C-PC due to an increase in electrostatic repulsion, which reduces aggregation and precipitation of compounds (Mogany et al. 2019). The most commonly isolated phycocyanin is derived from mesophilic organisms and is sensitive to physical factors such as light, temperature, pH and oxygen. The study carried out by Chaiklahan et al. demonstrated that 47 °C is the critical temperature for the stability of C-PC solutions (Chaiklahan et al. 2012). Therefore, the use of C-PC in food and other products is limited due to its sensitivity to heat treatment, which causes the blue pigment to precipitate and fade. This limits the use of phycocyanin in food products that require high-temperature processes such as cooking or sterilization (Pan-utai et al. 2018).

*Synechococcus* is a genus of thermophilic, unicellular cyanobacteria found in alkaline hot springs at 50–65 °C. Hot spring cyanobacteria have very stable enzymes that can catalyse enzymatic reactions under high temperature conditions. The adaptation mechanisms of thermophilic cyanobacteria to different stressful hot spring conditions make these organisms and their bioproducts of great value for biotechnology and various industrial applications (Cheng et al. 2020). C-PC from *Synechococcus* is one of the most stable proteins of this type (Liang et al. 2018). It has been shown to exhibit 90% stability after a 5 h incubation at 50 °C; it shows high stability at acidic pH and a good long-term stability. Therefore, it has been proposed to be considered as a thermostable substitute for *Spirulina* phycocyanin (Liang et al. 2018).

The aim of the study was to select and evaluate an efficient method for the separation and purification of phycobiliproteins in order to obtain a product that is not only highly pure, but also stable and can be used as a valuable bioproduct.

## Materials and methods

### Crude extract of phycobiliproteins

The crude extract of phycobiliproteins was obtained from *Synechococcus* sp*.* PCC 6715 bioreactor cultivation after biomass disintegration and protein extraction. The thermophilic strain *Synechococcus* sp. PCC 6715, originally isolated from Yellowstone National Park (USA), was purchased from the Collection of Cyanobacteria Institute Pasteur (Paris, France). Details of the cultivation in a laboratory helical-tube photobioreactor were presented in Gluszcz et al. (2018) and the biomass disintegration by alternating cycles of freezing and thawing was described in Klepacz-Smółka et al. (2020). The resulting product called PBP crude extract was then stored in a freezer and used in ongoing experiments.

### Purification procedure

The two-step method for the concentration and separation of the PBPs from their crude extract was investigated. Foam fractionation (FF) was performed followed by preparative chromatography. The experimental set-up for FF has been described in detail by Antecka et al. (2022). It consists of a glass column with a porous glass disperser at the bottom and a foam collector at the top. The condensate was collected in a Büchner flask connected to a low vacuum pump. Air was supplied by a compressor with a pressure reducing valve set at 2 bar. In each experiment, approximately 110 mL of crude extract of PBPs was added to the column, the air flow was set at 2.4 L/h and run until the foam could no longer reach the top of the column before collapsing. The resulting condensate was further purified by fast protein liquid chromatography (FPLC) using an ÄKTA Pure system (GE Healthcare). Four different columns HiTrap, Mono Q, Resource Q 1 and 6 mL were tested to select the one with the highest efficiency. Proteins were eluted with a linear gradient of 0 to 0.3 M NaCl in 10 mM Na-acetate buffer (pH 5.0) at a flow rate of 1, 4 or 6 mL/min, depending on the column tested. After separation the resulting fractions were pooled separately and stored at 4 °C for further investigations.

### Analytical methods

The concentration of C-PC and APC in the solutions was determined spectrophotometrically (BioTek EPOCH 2) by recording UV–Vis absorption spectra in the range from 250 to 800 nm, and calculated using the equations (Eqs. 1, 2) given by Bennett and Bogobad (1973). The maximum absorbance was measured for C-PC at 616 nm and at 652 nm for APC. All the spectrophotometric measurements of each sample were performed in triplicate.

1 $${C}_{C-PC}=\frac{{A}_{616}-0.474 \cdot{A}_{652}}{5.34} \left(\frac{mg}{mL}\right)$$

2 $${C}_{APC}=\frac{{A}_{652}-0.208\cdot{A}_{616}}{5.09}\left(\frac{mg}{mL}\right)$$

where *A* is the absorbance at a given wavelength.

The molecular weight (MW) of the phycobiliproteins was determined by sodium dodecyl sulphate–polyacrylamide gel electrophoresis in gradients of 4% and 15% (SDS-PAGE; Novex, Xcell SureLock Mini Cell, Invitrogen, Karlsruhe, Germany). Electrophoretic analysis was performed at a constant voltage of 120 V using 1xTGS as an electrode buffer. Samples were applied to the gel in a volume of 20 µL. After electrophoresis, the gel was stained and protein bands were visualized using a colloidal blue staining kit from Invitrogen (Karlsruhe, Germany); a low molecular weight protein calibration kit was used as a standard (MBI Fermentas, St. Leon Roth, Germany). Theoretical stripe masses were determined using QuantityOne software (Bio-RAD), based on the PageRuler 10–180 kDa standard (ThermoFisher Scientific).

### Purity and yield parameters

The purity ratio (P) which indicates the amount of C-PC or APC to other contaminating proteins was calculated from the spectroscopic absorbance at two wavelengths according to equations (Eqs. 3, 4) (Liu et al. 2005).

3 $${P}_{C-PC}=\frac{{A}_{616}}{{A}_{280}} (-)$$

4 $${P}_{APC}=\frac{{A}_{652}}{{A}_{280}} (-)$$

The recovery yields (Y) (Eq. 5) and purification factors (PF) (Eq. 6) were calculated as previously (Antecka et al. 2022).

5 $$Y=\frac{{C}_{p}\cdot {V}_{p}}{{C}_{c}\cdot {V}_{c}}\cdot 100 (\%)$$

6 $$PF=\frac{{P}_{p}}{{P}_{c}} (-)$$

where *C* is the concentration of PBP, *V* is the volume, the subscript* p* refers to the phase, in which PBP is concentrated and the subscript *c* refers to the crude extract introduced into the system.

### Stability investigations

The temperature stability of purified PBPs was determined by varying the cuvette temperature in the spectrophotometer (BioTek EPOCH 2) from 30 to 64 °C using an integrated Peltier element. For the measurements, samples were incubated for 5 h at different temperatures (30, 40, 50, 60, 64 °C) in acetic buffer (pH 5), and the residual activity was measured first every half hour and then every hour.

To estimate the optimal pH of the proteins, the pH of the samples was varied in the range from 3 to 12 and the concentration of PBPs was measured; pH stability was tested by storing the samples for 5 h in acetic buffers (10 mM), whose pH was changed to the assigned values of 3, 4, 5, 7, 9, 10, 11 and 12. All measurements were carried out at room temperature.

Long-term stability was measured during 30 days of storage at the following three temperatures: 4, 25 and 45 °C.

Photostability was determined by exposing the samples to a UV–VIS lamp (600 W, Lumatek,). UV A (3 W/m^2^), UV B (2.6 W/m^2^) and UV ABC (274.6 W/m^2^) lamps (Philips) were also used.

## Results

### Purification procedure

**Fig. 1:**
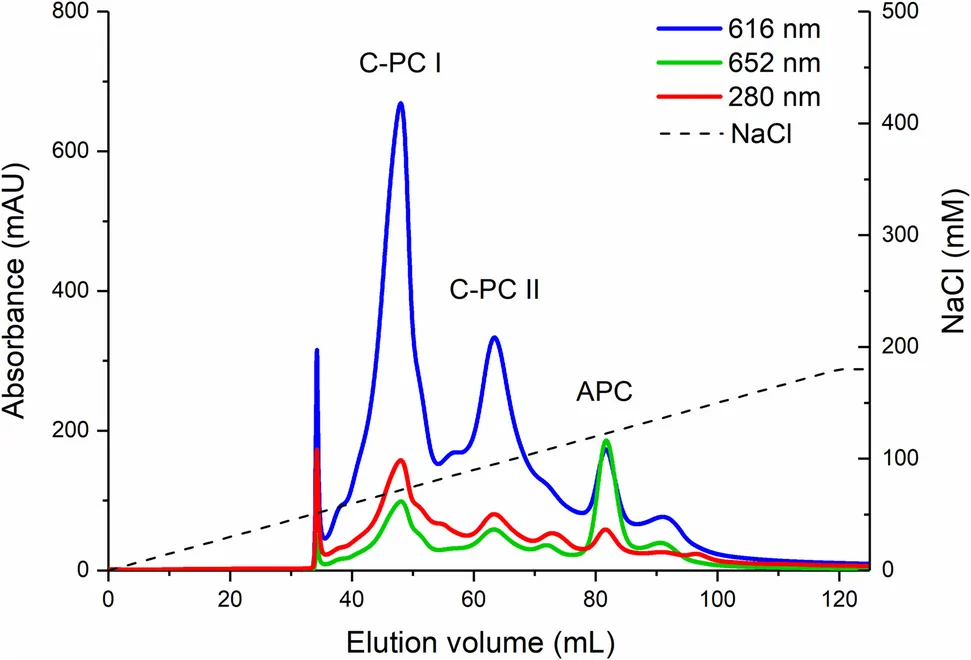
Chromatogram of phycobiliproteins from *Synechococcus* PCC 6715 obtained by anion exchange chromatography on a Resource Q column. Absorbance at 280, 616 and 652 nm (solid lines), NaCl gradient (dashed lines)

The crude extract of phycobiliproteins with the purities of 1.24 for C-PC and 0.445 for APC was subjected to two further steps of downstream processing. As previously demonstrated (Antecka et al. 2022), the foam fractionation process can be successfully used to concentrate C-PC from the crude solution. In addition, it does not require the addition of surfactants or pH adjustment, but only air, which greatly reduces the cost of operation. In this step, the recovery yielded over 47% and the purity of C-PC was increased by 1.36 times. The obtained C-PC had a purity of 1.69 and can be classified as a food or cosmetic colorant (Wu et al. 2016; da Silva Figueira et al. 2018), whereas, the purity of APC after the FF process was equal to 0.516 with the recovery of 17.4% and the purification factor of 1.16. The resulting condensate was then subjected to preparative liquid chromatography (FPLC), which is considered the most efficient method for separating C-PC from solution (Ledakowicz et al. 2024). Of the four columns tested, the highest purity of C-PC and its complete separation from allophycocyanin was obtained on a Q Resource column in a system with 10 mM acetate buffer, pH 5.0, and elution with 1 M NaCl in a 0–18% gradient. Total protein recovery was approximately 23.9% for C-PC and 8.60% for APC, while the purification factors were approximately 3.60 and 7.26, respectively. After a single pass through the Resource Q column and a relatively slow elution with a linear gradient, two separate fractions containing C-phycocyanin and one containing allophycocyanin were obtained, as shown by the two main peaks labelled C-PC I and C-PC II, and an allophycocyanin peak labelled APC (Fig. 1).

The two fractions of deep blue-colour C-PC and greenish-blue-colour APC were collected separately and subjected to stability testing. The results of the entire purification procedure are presented in tables (Table 1) for C-PCs and (Table 2) for APC. The protein recovery yields were 17.4% for C-PC I and 6.52% for C-PC II at a purity of 4.66 and 4.25, respectively and 8.60% for APC at a purity of 3.23.

**Table 1:** Purification of C-PCs from *Synechococcus* PCC 6715

Process/object			Volume (mL)	Conc. C-PC (mg/mL)	Protein (mg)	Purity (-)	Yield (%)	PF (-)
Crude extract			113	0.292	33.0	1.24	-	-
FF			17.0	0.919	15.6	1.69	47.3	1.36
FPLC	C-PC I	16.0		0.359	5.74	4.66	17.4	3.76
	C-PC II	12.0		0.179	2.15	4.25	6.52	3.43

**Table 2:** Purification of APC from *Synechococcus* PCC 6715

Process/object		Volume (mL)	Conc. APC (mg/mL)	Protein (mg)	Purity (-)	Yield (%)	PF (-)
Crude extract		113	0.0569	6.43	0.445	-	-
FF		17.0	0.0660	1.12	0.516	17.4	1.16
FPLC	APC	8.00	0.0691	0.553	3.23	8.60	7.26

### Characterization of the bioproducts obtained

Extensive work has been carried out to investigate the effect of temperature, pH, time and light on the stability of purified C-PCs and APC. The individual, detailed results are presented in the following subsections below.

### Temperature stability

**Fig. 2:**
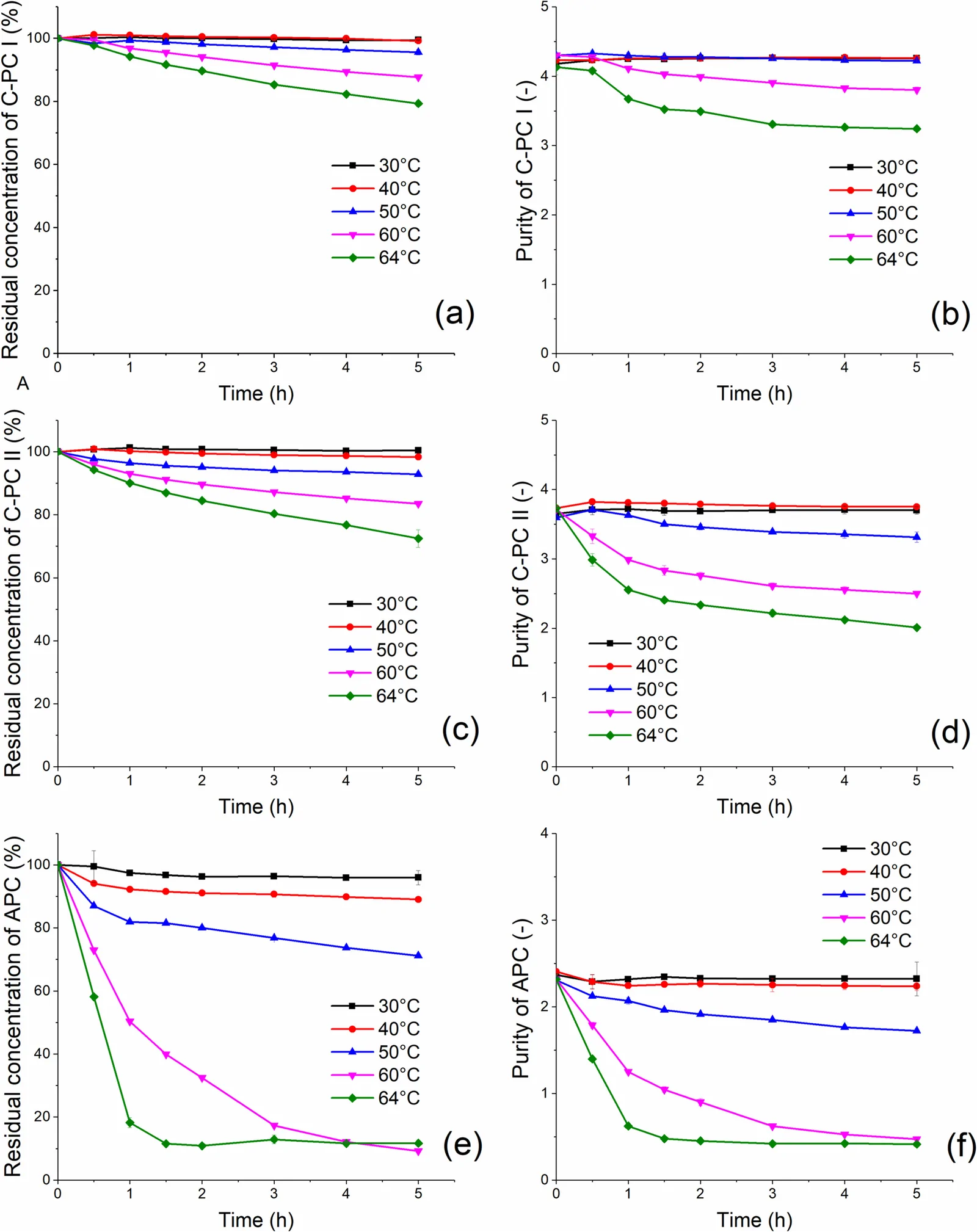
Stability and purity of C-PCs and APC during 5 h incubation at different temperatures; **a** stability of C-PC I, **b** purity of C-PC I, **c** stability of C-PC II, **d** purity of C-PC II, **e** stability of APC, **f** purity of APC. Measurements were performed in triplicate (standard deviations < 5%)

Temperature is one of the most important factors affecting the stability of pigment proteins present in cyanobacteria. Chentir et al. (2018) reported a dramatic decrease in the concentration of C-PC from *Spirulina* at temperatures above 50 °C. However, for C-PC from *Synechococcus* this critical factor does not seem to be as destructive as for C-PC from mesophilic species. In this experiment the purified PBPs were subjected to heat stress from 30 to 64 °C for 5 h. The results obtained are shown in Fig. 2.

Both fractions of C-PC (Fig. 2a, c) were found to be stable at temperatures of up to 50 °C, with no significant changes in the concentration values observed. However, when the temperature was increased from 40 to 64 °C, the residual concentrations of C-PC I after 5 h of incubation decreased from 99% at 40 °C to 96% at 50 °C, 88% at 60 °C and 79% at 64 °C. In addition, fraction C-PC I appears to be slightly more stable than fraction C-PC II, as the residual activity after 5 h at 64 °C was 79% instead of 72% for C-PC II. The degree of purity of the isolated C-PCs (Fig. 2b, d) varied with time, as did their concentration. During short-term exposure at temperatures as high as 60 °C, the thermal decomposition of C-PC is not as apparent, probably influenced by the helical secondary structure of the phycocyanin. Interestingly, the second pigment APC was much less stable than C-PC (Fig. 2e, f). Its concentration decreased slightly already at 40 °C, and after 5 h of incubation the residual activity was 89%, with rapid denaturation at temperatures above 60 °C.

### Optimum pH and stability

**Fig. 3:**
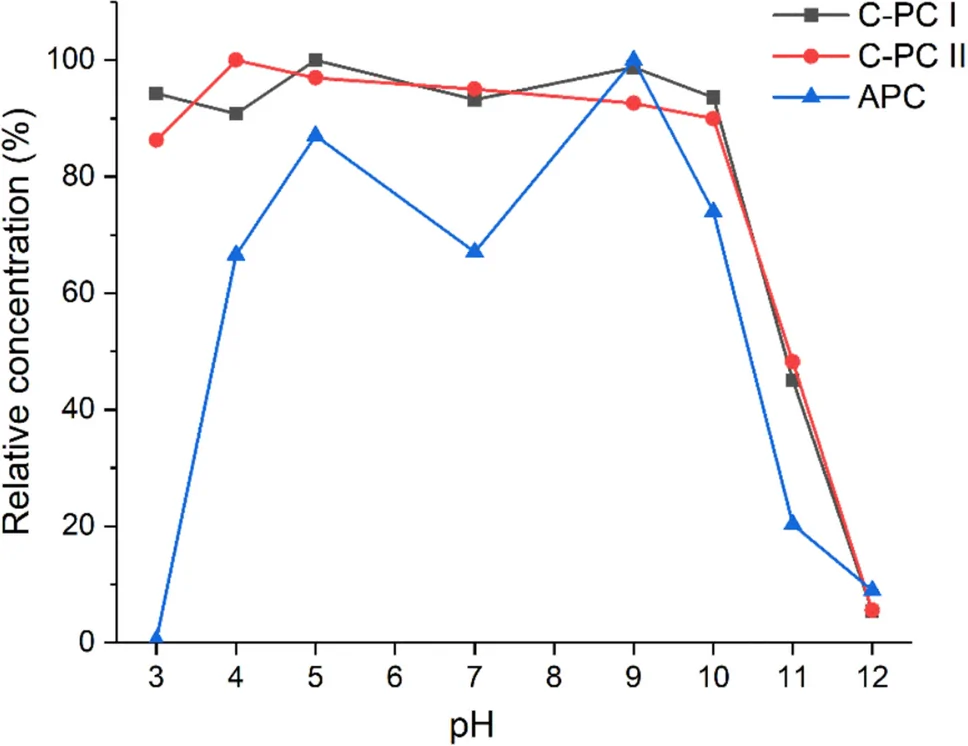
Effect of pH on the concentration of purified PBPs: C-PC I, C-PC II and APC. Measurements were performed in triplicate (standard deviations < 5%)

The pH of the solvent is also a critical factor in the application of bioproducts, as proteins are sensitive to changes in pH. Therefore, in order to determine the optimal pH range for purified PBPs, samples were placed in acetic buffers with different pH values ranging from 3 to 12. The results (Fig. 3) show that the C-PC remains unchanged over a wide range of pH from 3 to 10, only a strongly alkaline pH causes immediate discoloration of the pigment. In contrast, the second product, APC, is much more sensitive to pH changes, remaining unchanged only at pH 5 and 9. At pH values of 4, 7 and 10 the pigment concentration retained about 70% of its initial value, while pH 3 and 12 caused immediate protein degradation.

**Fig. 4:**
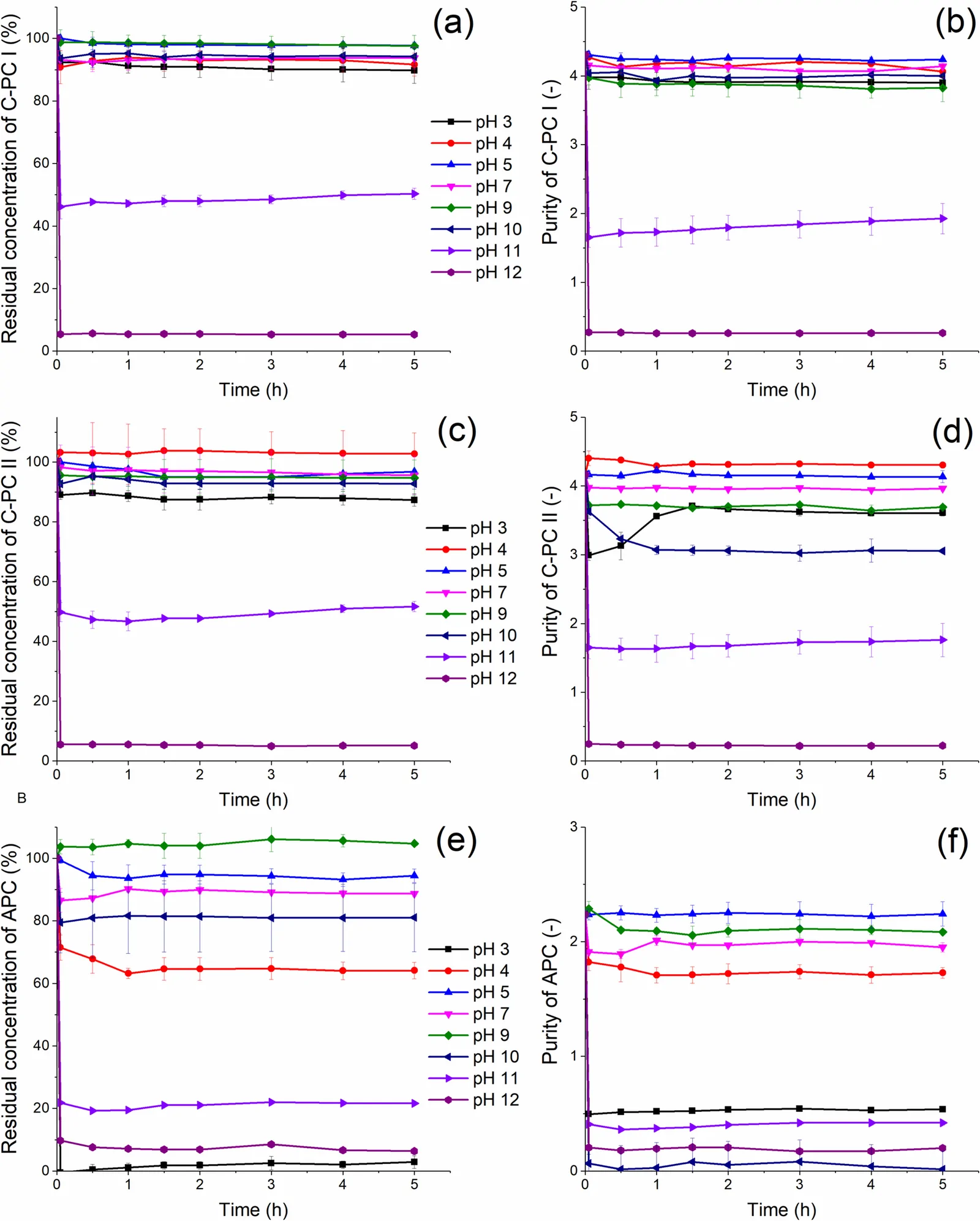
Stability and purity of isolated C-PCs and APC during 5 h incubation at different pH values; **a** stability of C-PC I, **b** purity of C-PC I, **c** stability of C-PC II, **d** purity of C-PC II, **e** stability of APC, **f** purity of APC. Measurements were performed in triplicate

Although knowing the optimum pH for a protein is very useful, from a practical point of view, its stability under given conditions is even more important. Therefore, to determine the stability of the PBPs, samples were incubated for 5 h at given pH values ranging from 3 to 12. The results for C-PCs (Fig. 4a, c) show that both its fractions remain stable at pH 3 to 10. In alkaline solutions (pH 11), the concentration of C-PC immediately decreased by about 50% and then it remained stable for 5 h, while pH 12 caused an immediate decolorization of the pigment. As with the temperature stability, fraction C-PC II was more sensitive to pH changes than fraction C-PC I.

The purity of both fractions of C-PC (Fig. 4b, d) varied similarly to their concentration during incubations at different pH values, confirming the absence of other proteins in the samples. APC appeared to be much more pH-dependent, as its concentration remained unchanged only at pH 5–9 (Fig. 4e, f), whereas strongly acidic (pH 3) or alkaline (pH 11–12) pH induced rapid pigment degradation. However, all the results obtained suggest that PBPs from *Synechococcus* are more pH stable than C-PC from *Spirulina*, which is only stable at pH 5–7.5 when the hexameric form predominates (Pez Jaeschke et al. 2021).

### Long-term stability

**Fig. 5:**
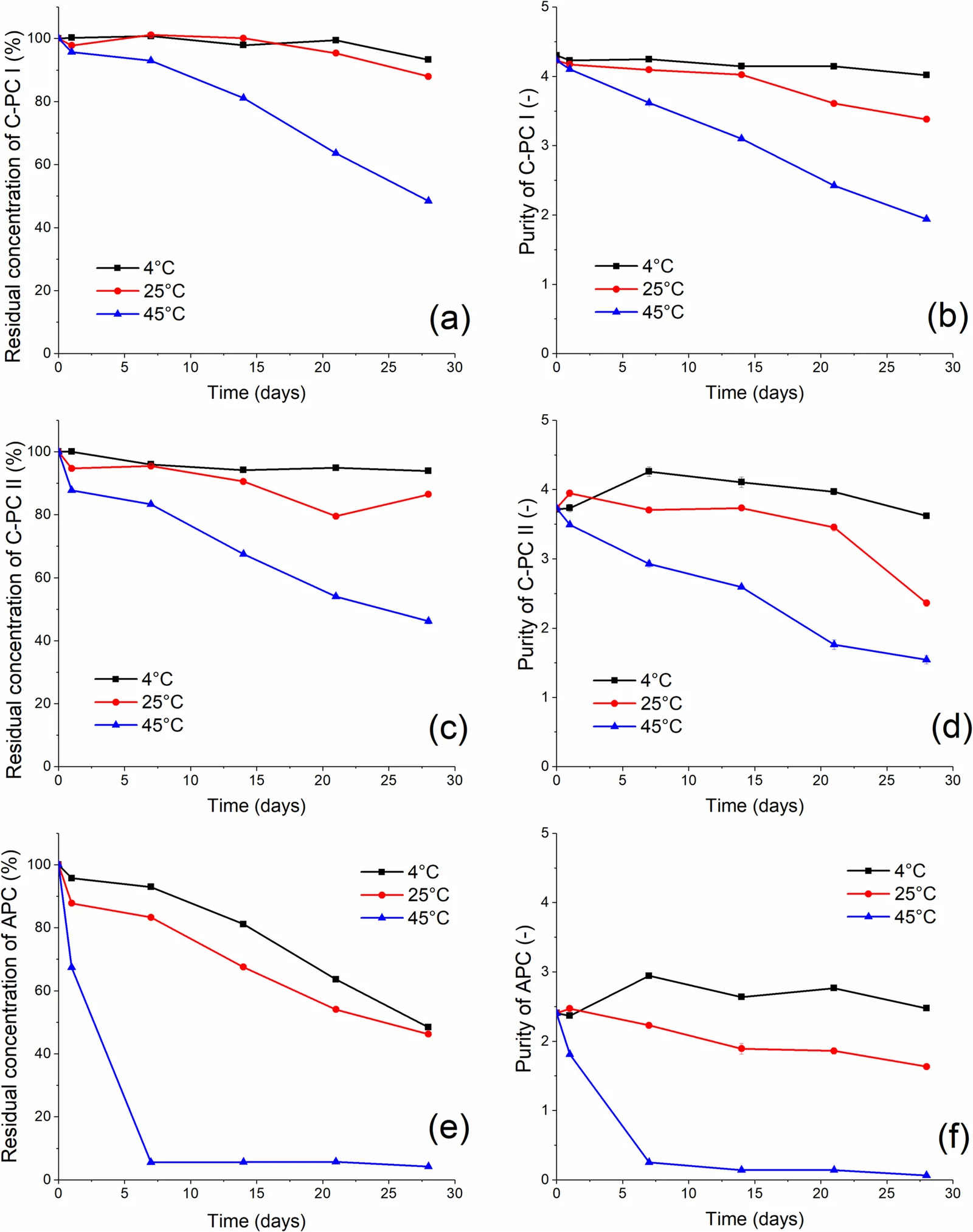
Long-term stability and purity of isolated C-PCs and APC during one month of storage at three different temperatures; **a** time stability of C-PC I, **b** purity of C-PC I, **c** time stability of C-PC II, **d** purity of C-PC II, **e** time stability of APC, **f** purity of APC. Measurements were performed in triplicate (standard deviations < 5%)

Appropriate storage conditions are another parameter that guarantees the preservation of the valuable properties of the proteins. Therefore, the long-term stability of purified PBPs and the effect of three different temperature storage conditions were investigated and the results are shown in the figures (Fig. 5).

The results (Fig. 5a, c) show little difference between the C-PC fractions, C-PC I was stable after 1 month of storage at 4 and 25 °C and the C-PC II only at 4 °C. The concentration of both fractions decreased significantly when stored at 45 °C. Interestingly, the purity of isolated C-PCs (Fig. 5b, d) remains unchanged only at 4 °C. This proves that the lower temperature helps maintain the stability of the phycocyanin for a longer period of time. For comparison, in the case of C-PC from *Spirulina* even ambient temperature caused a 20% loss in concentration after 10 days of incubation (Pez Jaeschke et al. 2021).

In the case of APC (Fig. 5e, f) a decrease in both concentration and purity was observed regardless of temperature, but at 45 °C APC was completely inactivated after 6 days of incubation. At temperatures 4 and 25 °C, a systematic decrease in APC concentration was observed, so that after 30 days of incubation the residual concentrations were approximately 50%. Interestingly, the purity was almost unchanged at 4 °C, indicating a similar decrease in both absorbance values, at 652 and 280 nm.

### Photostability

**Fig. 6:**
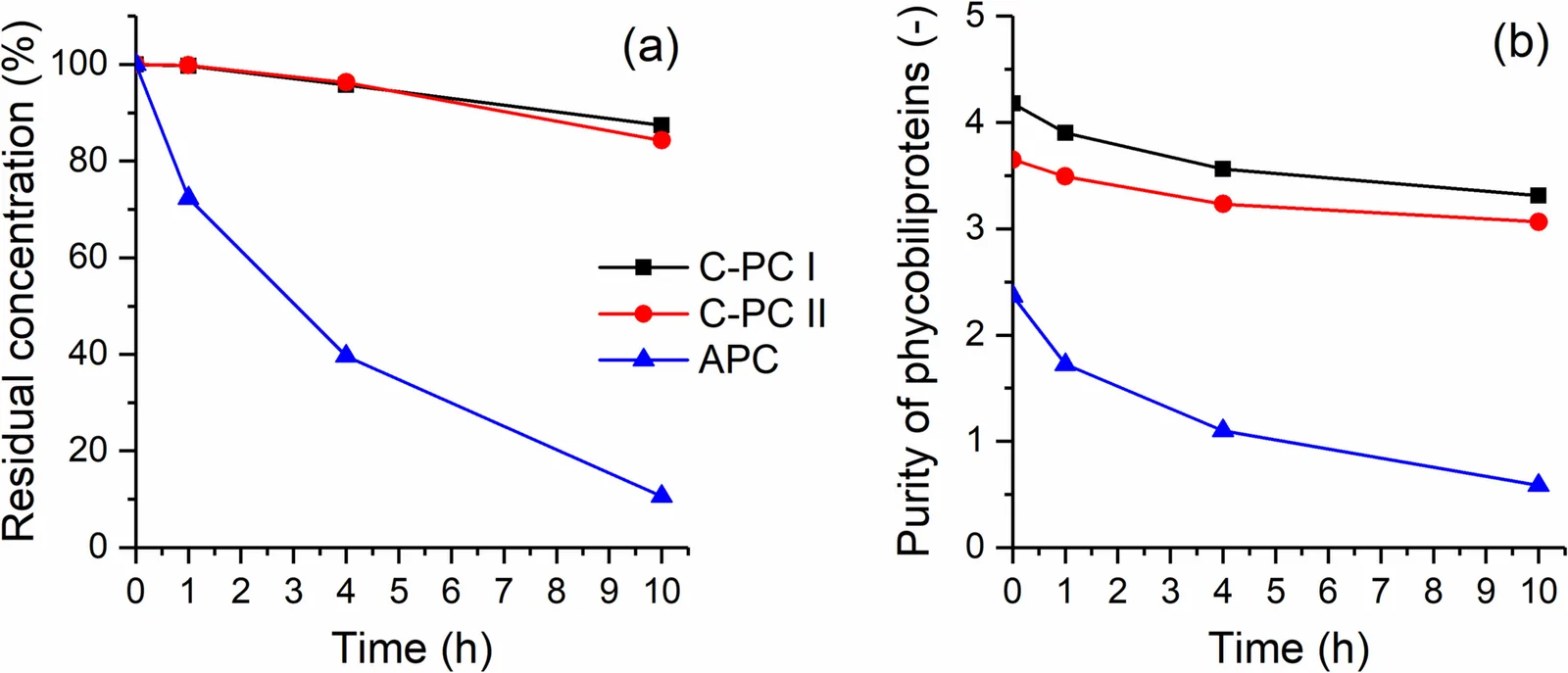
Photostability of purified PBPs during 10 h of light exposure in front of the UV–VIS lamp (600 W); **a** residual concentrations of PBPs, **b** purity of PBPs. Measurements were performed in triplicate (standard deviations < 5%)

With respect to light exposure (Fig. 6), a large difference was also observed between the C-PC fractions and the APC. APC was much more sensitive to photochemical degradation than C-PC. After 10 h of exposure C-PCs still had about 86% of their initial concentration, while APC had only about 10%.

### Molecular weight

**Fig. 7:**
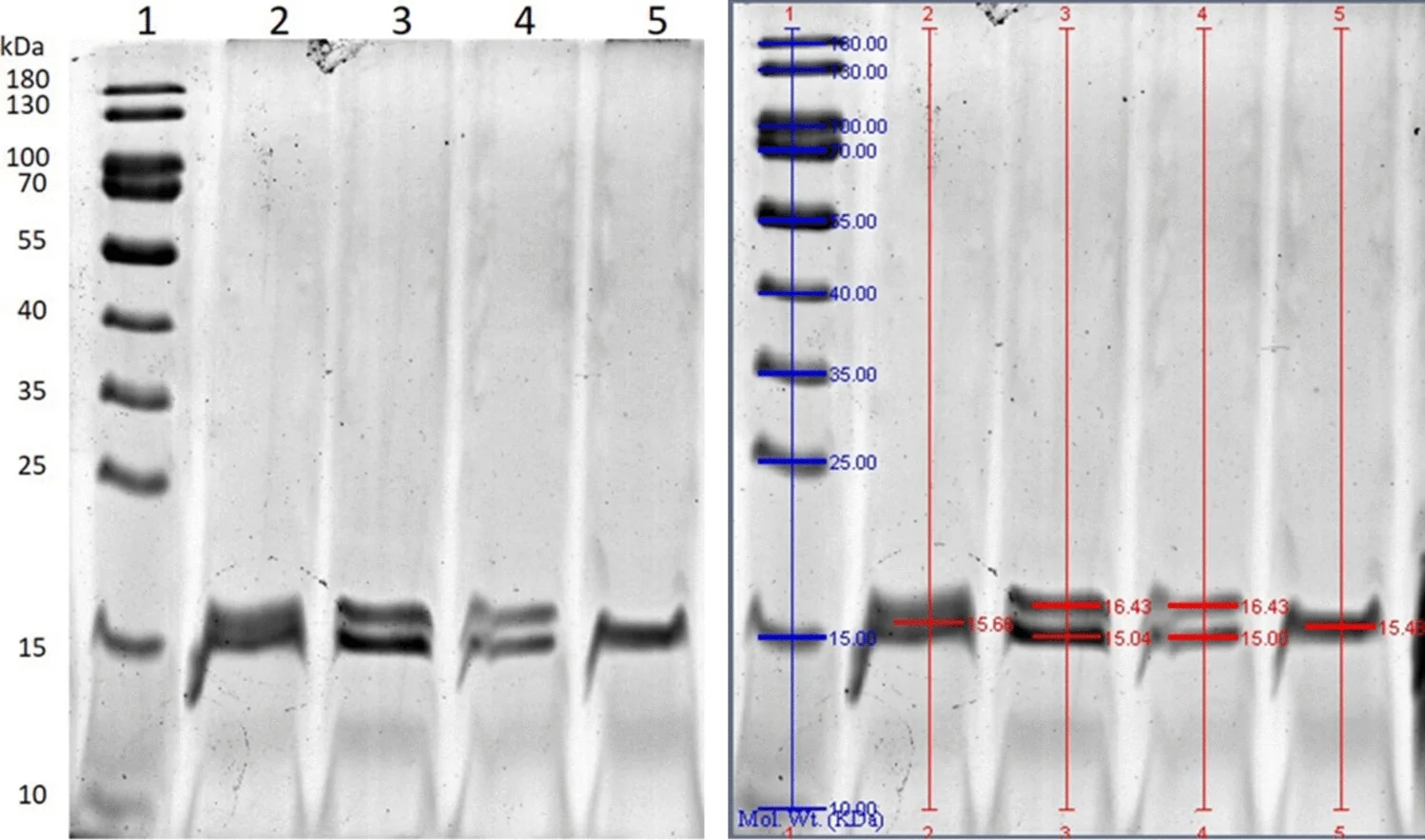
SDS PAGE of *Synechococcus* PCC 6715 phycobiliproteins after two-step of purification. Line 1. Ladder: PageRuler 10–180 kDa (ThermoFisher Scientific); line 2. Crude extract; line 3. C-PC I; line 4. C-PC II; line 5. APC

SDS-PAGE analysis of purified PBPs (Fig. 7) shows that both isolated phycocyanin fractions contain α-subunits of 15.0 kDa and β-subunits of 16.4 kDa, confirming their identity by native-PAGE. The molecular weight of allophycocyanin is 15.5 kDa. These experimental results indicated that the purification procedure used produced reliable, highly pure PBPs.

## Discussion

Currently a lot of research is focused on finding and developing novel, cost-effective and efficient methods for the separation and recovery of bioproducts with satisfactory selectivity for the desired product and ensuring its further stability. Phycocyanin, as a target product from cyanobacteria, with numerous advantages is a frequent subject of research related to its downstream processing methods (Patil et al. 2006; Moraes and Kalil 2009; Brião et al. 2020). The idea is to obtain C-PC with an appropriate degree of purity using a minimum number of purification steps (da Silva Figueira et al. 2018). In this report, we have documented that the purification of phycobiliproteins can be performed in a simple two-step process using a combination of foam fractionation and single-shot ion exchange chromatography. In contrast to chromatography, the use of foam fractionation for protein purification is much less common and, to the best of our knowledge, has not been described in the literature for the separation of PBPs prior to our reports (Antecka et al. 2022, 2024; Ledakowicz et al. 2024). However, the method has a number of advantages that make it a feasible process. Firstly, it is carried out under mild conditions for biological molecules and it is suitable for dilute solutions (Uraizee and Narsimhan 1990). It is also cost effective and environmentally friendly as it does not require the use of sophisticated equipment or chemicals, only inert gas (Burghoff 2012). Finally, foam fractionation tends to be selective for proteins since they are mostly amphiphilic compounds that are concentrated at the liquid–gas interface (Blatkiewicz et al. 2017). As demonstrated in this work, the foam fractionation process can be successfully used to concentrate C-PC from the crude solution. Furthermore, it does not require the addition of surfactants, as is the case for other bioproducts (Linke et al. 2007), nor temperature or pH adjustment, which significantly reduces the cost of implementation (Antecka et al. 2022, 2024). This time the process resulted in a more than sixfold reduction in volume and a more than threefold increase in concentration, as well as a 1.36-fold increase in phycocyanin purity. The second, chromatographic separation step yielded two phycocyanin fractions with purities of 4.66 and 4.25, and an allophycocyanin fraction with a purity of 3.23. Such a high purities achieved in just a single chromatographic step are relatively rare. For comparison, Moraes and Kalil (2009) obtained two fractions of C-PC from *S. platensis* LEB 52 with purities of 4 and 3.88, using a two-step process with chromatography preceded by precipitation. The use of other processes such as ultrafiltration (Brião et al. 2020) or even multi-step separation (da Silva Figueira et al. 2018) usually results in food or reagent purity levels, below 3.9 (Hsieh-Lo et al. 2019). However, recently Safaei et al. (2019) reported the production of a C-PC pigment from a local strain, *Limnothrix* sp. NS01, with a purity of 5.26 during a four-step purification process, confirming that the design and combination of processes can achieve very high purity of the final product.

Another issue is the stability of the resulting bioproduct, which is known to be highly dependent not only on the strain and culture conditions, but also on the factors such as light, low pH values, strong ionic strengths or high temperatures (Hsieh-Lo et al. 2019). Therefore, much effort has been put into investigating stability of the PBPs under different, including adverse environmental conditions (Nowruzi et al. 2022) or finding the methods to improve it (Hsieh-Lo et al. 2019). Since this study the two *Synechococcus* phycocyanin fractions have proven to be stable over a wide range of temperatures from 4 to 50 °C, and at 60 °C the concentration of C-PC I during a 5 h incubation still retains about 87% of the initial value. Therefore, the obtained results are even slightly better than those previously reported for this strain, where 90% of stability was reported after 5 h of incubation at 50 °C and 65% at 65 °C under the same conditions (Liang et al. 2018). The results confirm that besides the temperature, pH is the second most important factor affecting PBPs stability (Chaiklahan et al. 2012). This is because due to ionic strength of the solvent, pH directly affects the solubility of the protein and its structure. However, the purified C-PCs from *Synechococcus* remain stable at pH 3 to 10, which is a much wider range than the 6–8 indicated by Su et al. (2014) or Patil and Raghavarao (2007), who mentioned that C-PC is unstable at pHs below 5 and above 8. In this report, only strong alkaline solutions (pH > 11) caused immediate decolorization of the pigment. The observed differences in the stability and physicochemical properties of C-PC I and C-PC II may suggest that the C-PC produced by *Synechococcus* is not homogeneous. However, the literature report (Liang et al. 2018) for this strain suggests a single protein. To be sure, genetic testing would be required. From our study, the SDS-PAGE analysis performed indicates that both C-PC fractions contain α- and β-subunits with the same MW characteristic for C-PC from *Synechococcus* (Liang et al. 2018). Thus, the separation of C-PC I and C-PC II observed in the chromatographic step is more likely due to differences in their ionic strength, resulting in a slightly different affinity between the fractions and the resin. Similar behaviour was also described by Amarante et al. (2020), where the number of C-PC fractions depended on the method parameters, but the differences between the fractions were not investigated. From this study, C-PC I, which has a higher concentration and purity ratio, is less sensitive to temperature and pH changes and its purity is slightly more stable. Surprisingly, the second phycobiliprotein, APC appeared to be much more pH-dependent, as its concentration remained unchanged at pH 5–9, whereas strongly acidic or alkaline pH induced rapid pigment degradation. It should be added, that all tests were carried out without a stabilizing agent, which is recommended to protect the molecules (Pez Jaeschke et al. 2021). In addition, C-PC is much more resistant to light than APC, its photochemical stability being 86% of the initial concentration after 10 h of incubation under the UV–VIS lamp. These results will enrich the review of the light stability of the PCs made by Nowruzi et al. (2022), where it is stated that light strongly affects the stability of PBPs, as the storage of the PC in light reduces its half-life.

In conclusion, the main achievement of this work has been to obtain a bioproduct with the highest analytical purity class (purity > 4), which allows it to be used in the food, cosmetic, medical and pharmaceutical industries and which is stable over a wide temperature and pH range without the addition of a stabilizing agent. This has been achieved by using an innovative process: initial concentration of the crude phycocyanin solution using the foam fractionation method and further separation using a single-stage ion chromatography method. The most suitable storage conditions for the C-PC product, which ensure that the concentration and purity remain unchanged for at least 30 days, are temperatures of 4 °C and pH 4–5.

## Data Availability

All data generated or analysed during this study are included in this published article or will be available on request.
